# Supplementary material for: The TOR‐dependent phosphoproteome and regulation of cellular protein synthesis
Source: EMBO J. 2021 Jul 23;40(16):e107911. doi: 10.15252/embj.2021107911 (PMC8365262; doi:10.15252/embj.2021107911)
Supplement: Supplementary file 2 — Expanded View Figure PDF [file EMBJ-40-e107911-s001.pdf]

Expanded View Figures

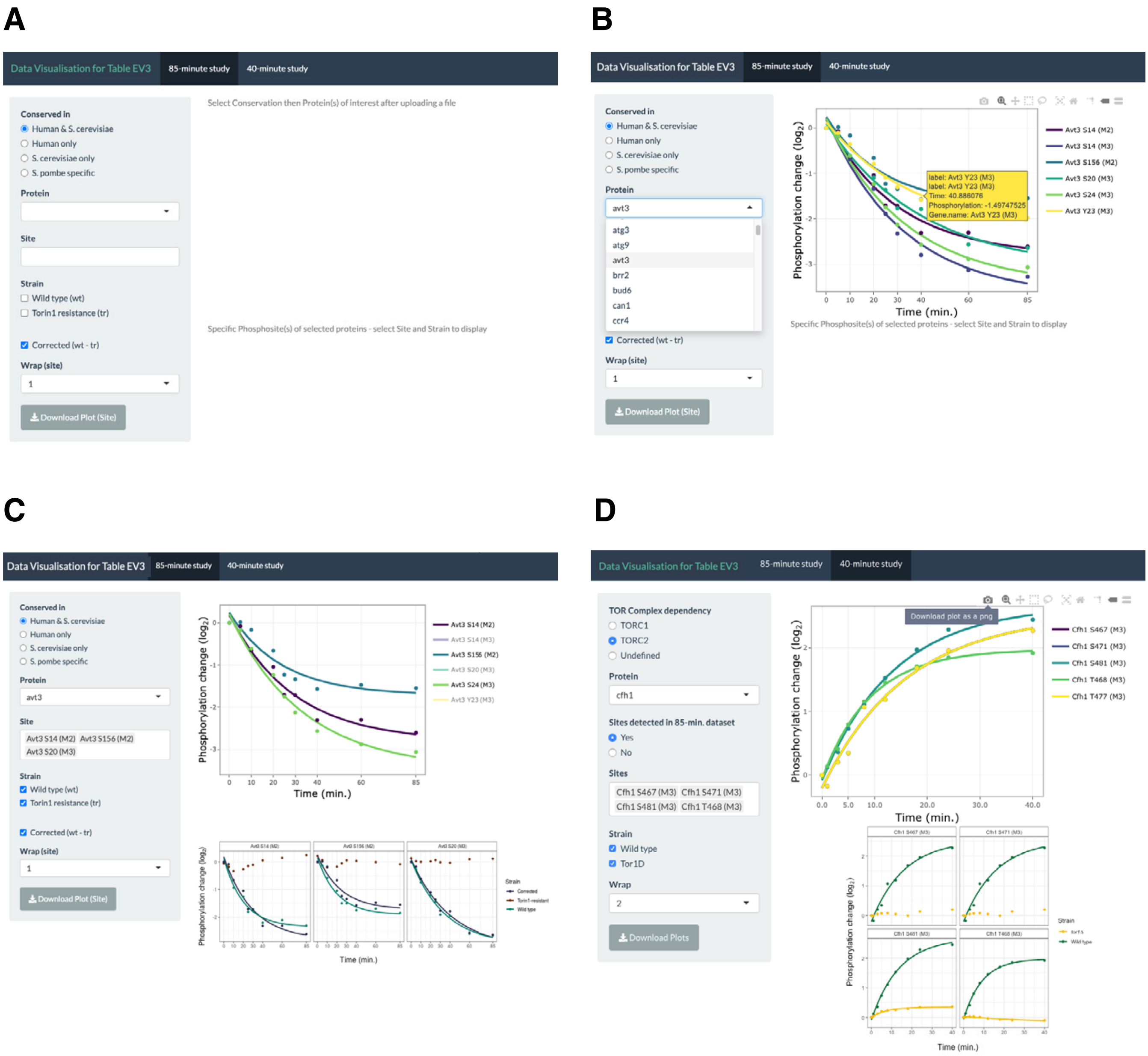

Figure EV1. Illustrated example for data visualisation tool.

Example interface of the data visualisation tool for visualising the phosphorylation data of the 908 phosphosites (Table EV3) from the two phosphoproteomics time courses. Information regarding file upload, data filtering and subsequent visualisation as detailed in Materials and Methods.

- A Initial interface of the first “85-min study” tab upon opening the application from the given link.
- B Demonstration of initial filtering of protein list based on conservation and subsequent display of graph with all phosphosites in top panel.
- C Illustration of individual phosphosite and specification of strain dataset to display in lower facet panels.
- D Interface of the second “40-min study tab” interface with additional filtering criteria on sidebar panel and demonstration of the “Wrap” and “Download” functions.

© 2021 The Authors

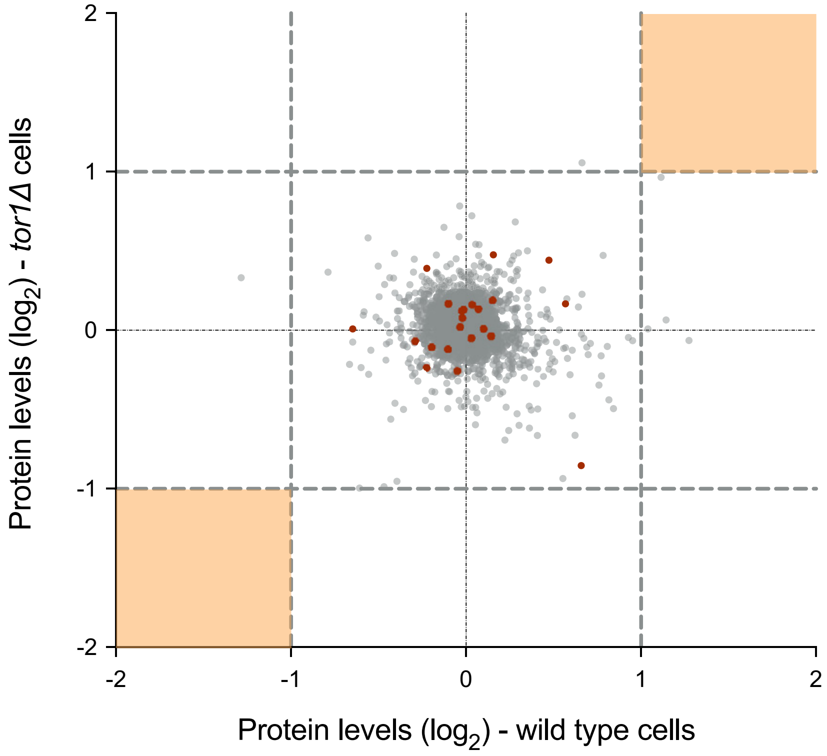

Figure EV2. Relative protein expression levels in wild type and *tor1Δ* after 40 min of Torin1 treatment.

Scatter plot of the relative expression levels of individual protein in wild type and *tor1Δ* cells from the 40-min proteomic time course experiment. Twofold thresholds are represented by grey dashed lines for either direction in the respective strains. Orange shaded areas indicate the expected regions with more than 2-fold changes in relative protein expression levels in both wild type and *tor1Δ* cells, of which none were detected in the study. Points highlighted in red indicate the 23 proteins showing over 1.5-fold difference in expression levels between wild type and *tor1Δ* before Torin1 treatment. But the relative expression levels of these proteins remain within a 2-fold range upon inhibition of TOR activity inhibition.

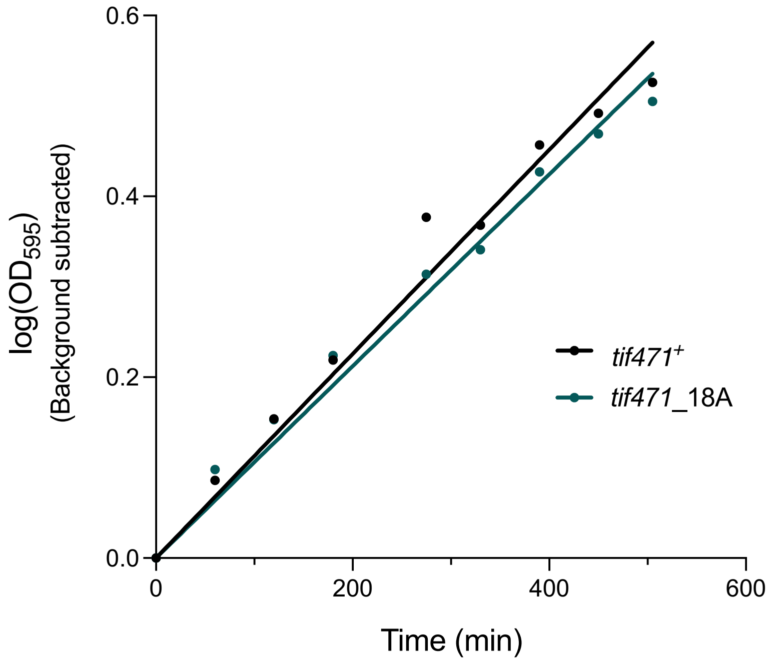

Figure EV3. Relative growth rates in *tif471*_18A mutant is comparable to wild type cells.

Representative plot of the relative growth rates as measured by the change in optical density (OD_{595}) over time for *tif471*_18A mutant and control (*tif471*$^{+}$) cells. Values are background subtracted and gradients of the regression lines were 0.0011 for both strains, fitted using simple linear regression on Prism 9.

© 2021 The Authors
